# Correction: Feeling connected but dissimilar to one’s future self reduces the intention-behavior gap

**DOI:** 10.1371/journal.pone.0321799

**Published:** 2025-04-04

**Authors:** Benjamin Ganschow, Sven Zebel, Jean-Louis van Gelder, Liza J. M. Cornet

The first author, Benjamin Ganschow, is incorrectly noted as the corresponding author. The correct corresponding author is Jean-Louis van Gelder. Jean-Louis van Gelder’s email address is: j.vangelder@csl.mpg.de.

In the Analytical strategy subsection of Results, there is an error in the second sentence of the first paragraph. The correct sentence is: Second, we predicted that the increase in the future self-continuity domains would be larger in the Imagine and Embodied-VR perspective taking exercise compared to the Imagine-IV or Imagine-VR exercises.

In the Analytical strategy subsection of Results, there is an error in the third sentence of the second paragraph. The correct sentence is: The second was an ANCOVA multiple mediation analysis that calculates the direct effect of condition on intention-behavior rate, the direct effect of the within-person change in future self-continuity domains on the intention behavior-rate, and thirdly the indirect effect of the within person change that explains the variation from the condition on the intention-behavior rate. [41].

In the Mediation of conditional effect through future self-domains subsection of Results, there is an error in the first sentence. The correct sentence is: Previous analysis found neither a conditional direct effect on the outcome (intention-behavior rate) nor on the mediators (future self-continuity domains), and therefore also no indirect effect (see online mediation results).

In the Discussion section, there is an error in the second sentence of the first paragraph. The correct sentence is: In this study, we aimed to reduce the intention-behavior gap by strengthening participants’ future self-continuity domains.

In the Imagination, embodiment, and the intention-behavior rate subsection of Results, there is an error in the first, fourth and sixth sentences of the third paragraph. The correct first sentence is: The main limitation to this study is our study may be underpowered to detect the effect size that our exercises would need to manipulate the intention-behavior rate. The correct fourth sentence is: This result is also seen in other studies [12, 14, 31] and future research should determine sample size with {} smaller effect sizes. The correct sixth sentence is: Lastly, we had two practical constraints that limited interpretation of our conclusions.
