# Supplementary figures and images for: Feeling connected but dissimilar to one’s future self reduces the intention-behavior gap
Source: PLoS One. 2024 Jul 23;19(7):e0305815. doi: 10.1371/journal.pone.0305815 (PMC11265703; doi:10.1371/journal.pone.0305815)

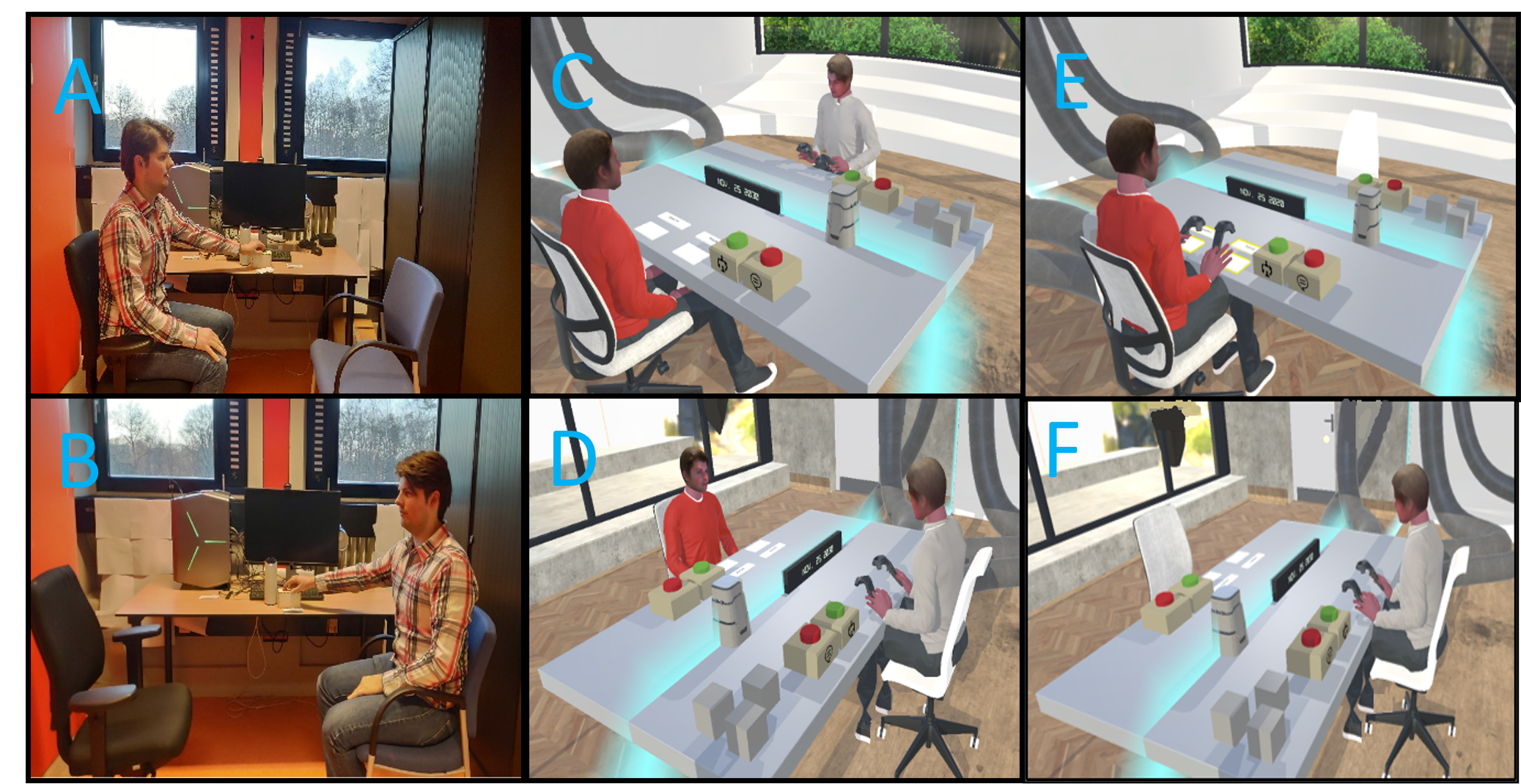

Supplement: S1 Fig — Note. Participants in Panels A and B are in the Imagine-IV, C and D in the Imagine and Embodied-VR and E and F in the Imagine-VR condition. Reprinted with permission from Benjamin Ganschow under a CC BY license, original copyright 2023. (TIF) [file pone.0305815.s001.tif]

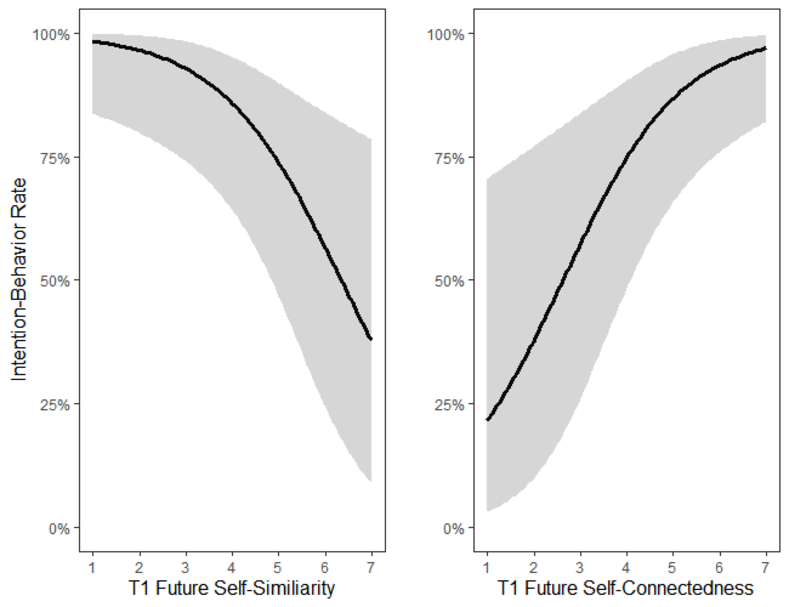

Supplement: S2 Fig — Note. Intention-behavior rates were predicted while controlling for T0 future self-continuity domains and VVIQ. Shaded areas represent 95% confidence intervals. (TIF) [file pone.0305815.s002.tif]
