# Supplementary material for: Feeling connected but dissimilar to one’s future self reduces the intention-behavior gap
Source: PLoS One. 2024 Jul 23;19(7):e0305815. doi: 10.1371/journal.pone.0305815 (PMC11265703; doi:10.1371/journal.pone.0305815)
Supplement: S1 Appendix — (DOCX) [file pone.0305815.s003.docx]

Appendix A – Writing Assignment

This question is adapted from the ‘best possible selves’ prompt from (King, 2001)

“Please try to imagine that in the next 10 years, you can achieve whatever you want.

There are no limits, no constraints (for example when it comes to energy, money, and knowledge). You can do and become everything you ever wished for. Perhaps you would like to run your own business, write a successful book, build your own house, or become a nomad traveling around the world. Think of what you want to do or become without any constraints. Again, everything is possible... you just must let go of limitations that you have in mind. This might sound like a difficult exercise, but once you think of something you would really like to do or become, your thoughts will start to flow. Take at least five minutes and write down one or multiple things you wish, dream, or hope to attain in the next 10 years. Please keep on writing (in English) and aim for a paragraph or two. Try to remember, everything is possible. If everything is possible, then in the upcoming years, I would like to. . .”
