# Supplementary material for: Feeling connected but dissimilar to one’s future self reduces the intention-behavior gap
Source: PLoS One. 2024 Jul 23;19(7):e0305815. doi: 10.1371/journal.pone.0305815 (PMC11265703; doi:10.1371/journal.pone.0305815)
Supplement: S2 Appendix — (DOCX) [file pone.0305815.s004.docx]

Appendix B – Perspective Taking Exercise Questions

| Tutorial | Experiment |
| --- | --- |
| 1. Future self, what did you have for breakfast? | 1. Future self, how are you feeling today? |
| 1. Future self, do you eat that every day? | 1. Future self, what are you celebrating? Who is coming? What are you guys going to do? |
| 1. Future self, where are you going on vacation this year? | 1. What does it feel like to finally achieve this? |
| 1. Future self, how much will this vacation cost? | 1. Future self, what did you do or change in the last years that helped you achieve this? |
|  | 1. What were some difficult experiences you overcame and how did you deal with them? |
|  | 1. What one lesson did you learn from these experiences? |
|  | 1. Future self, what is one daily habit that I should begin to achieve this goal? |
|  | 1. Future self, what are the best parts about being you? 2. Future Self, are you happy? |
|  | 1. Think of a question you would like to ask your future self and ask it. Start the question with: Future self… |
