# Supplementary material for: Feeling connected but dissimilar to one’s future self reduces the intention-behavior gap
Source: PLoS One. 2024 Jul 23;19(7):e0305815. doi: 10.1371/journal.pone.0305815 (PMC11265703; doi:10.1371/journal.pone.0305815)
Supplement: S3 Appendix — (DOCX) [file pone.0305815.s005.docx]

Appendix C – Technical Details

In the *in vivo* condition, the buttons were linked through an Arduino Micro microcontroller to a record and playback script created in the Processing programming language version 3.5.1 (Processing, 2019) that was adapted from recording and playback scripts from the minim library version 2.0.2 (Free Software Foundation, 2007). Audio was captured through ZOOM H4n Pro and recorded through Audacity version 2.3.1 (Audacity Team, 2019).

The virtual environment and avatar creation software was developed by Studio Barbaar (<https://studiobarbaar.com/>) in Unity and closely resembled the *in vivo* setup. The digital photos were taken with a Logitech C270 HD webcam. The VR environment was displayed through an HTC VIVE head-mounted display controlling head movements, with a field-of-view of 110°, and a resolution of 1080 x 1200 pixels per eye displayed at 90 Hz; two controllers providing control over arms and torso; and two base stations that allowed movement in 360°. The computer generating the virtual environments was an Alienware Aurora R7 running Windows 10 through an Intel i7-8700 CPU at 3.2 GHz, with a NVIDIA GeForce GTX 1080 Ti graphics card and 32 GB of RAM. The audio was captured as in the *in vivo* condition.
