# Supplementary material for: Feeling connected but dissimilar to one’s future self reduces the intention-behavior gap
Source: PLoS One. 2024 Jul 23;19(7):e0305815. doi: 10.1371/journal.pone.0305815 (PMC11265703; doi:10.1371/journal.pone.0305815)
Supplement: S4 Appendix — (DOCX) [file pone.0305815.s006.docx]

Appendix D – Behavior Selection Questions

Part of working towards your future self are the habits, like studying, eating, and exercising, that we do everyday. We want you to choose a habit to start! Do you remember the question that you asked yourself about starting a new habit? Take a few moments and think of a few things that you can and want to do in your daily life that would benefit your future self. If it is something you are already doing, think of something additional to that goal (e.g., "more X" or "longer Y"). If you have any questions, ask the researcher. If you are having trouble finding one, here are some helpful habits that are popular in general. https://medium.com/coach-me-app/top-100-habits-to-track-in-2018-4940bf459d5
After you choose three habits, we will evaluate them on time, importance and energy and choose one to begin.

Q1 What habits have you decided that you can and want to do?

- Habit 1 ________________________________________________
- Habit 2 ________________________________________________
- Habit 3 ________________________________________________

Q2 How often must you do these habits every week?

|  | Once a week (1) | Twice a week (2) | Three days (3) | Four days (4) | Five days (5) | Six days (6) | Everyday (7) |
| --- | --- | --- | --- | --- | --- | --- | --- |
| Habit 1 |  |  |  |  |  |  |  |
| Habit 2 |  |  |  |  |  |  |  |
| Habit 3 |  |  |  |  |  |  |  |

Q3 How many minutes does this habit generally take to do?

Habit 1: Free text

Habit 2:

Habit 3:

Q4 How important is this habit to your future goals and well-being?

|  | Not at all | Low importance | Slightly important | Neutral | Moderately important | Very important | Extremely important |
| --- | --- | --- | --- | --- | --- | --- | --- |
| Habit 1 |  |  |  |  |  |  |  |
| Habit 2 |  |  |  |  |  |  |  |
| Habit 3 |  |  |  |  |  |  |  |

Q5 After the evaluation, what habit would you like to start?

________________________________________________________________
