# Supplementary material for: Feeling connected but dissimilar to one’s future self reduces the intention-behavior gap
Source: PLoS One. 2024 Jul 23;19(7):e0305815. doi: 10.1371/journal.pone.0305815 (PMC11265703; doi:10.1371/journal.pone.0305815)
Supplement: S2 File — (DOCX) [file pone.0305815.s008.docx]

**Supplementary Materials 2**

Robustness check excluding the 15 participants who whose behavioral intention differed from T1 and T2 while also stating they followed through on their intention. Eight of these excluded participants were from the Imagine-IV, two from the Imagine-VR, and five from the Imagine and Embodied-VR. A chi-square test of independence found no statistically significant differences between the frequency of same and different of the three conditions, *X^2^*(2, N = 75) = 4.77, *p* = .092.

|  | Imagine-IV | Imagine-VR | Embodied-VR |
| --- | --- | --- | --- |
| Same | 13 | 18 | 20 |
| Different | 8 | 2 | 5 |
| Prop. Different | .38 | .1 | .2 |

**The Intention-Behavior Rate**

At T2, out of 75 participants, 53 (71%) responded that they had begun their behavioral intention. Additionally, 51 (68%) of the 53 who started the behavior also intended to continue afterwards.

**Mediation Analyses**

**Condition on Intention-Behavior Rate**

The proportion between conditions of the intention behavior rate was 68% for Imagine-IV, 71% for Imagine-VR, and 64% for Imagine and Embodied-VR. We ran a logistic regression of the direct effect of condition on the intention-behavior rate while controlling for imaginative ability and future self domains at T0. In comparison to the Imagine-IV condition, conditional results are the same as in main analysis and show no evidence that the Imagine and Embodied-VR or the Imagine-VR condition improving the behavior-intention rate (see Table 2). On difference is that future self vividness at T0 was predictive of the intention-behavior rate (b = 0.45, Z = 4.24, *p* = .040, OR =1.57)

**Condition on Future Self Domains**

Same results as reported in main analysis.

**Table 1.**

*Means and Standard Deviations of the Future Self Domains at* *T0 and T1.*

|  |  |  | T0 | | T1 | |
| --- | --- | --- | --- | --- | --- | --- |
|  | Condition | N | Mean | SD | Mean | SD |
| Connected | Imagine-IV | 19 | 4.5 | 1.8 | 5.0 | 1.4 |
|  | Imagine-VR | 29 | 4.0 | 1.7 | 4.8** | 1.4 |
|  | Imagine and Embodied-VR | 28 | 4.4 | 1.8 | 4.5 | 1.6 |
| Similarity | Imagine-IV |  | 3.8 | 1.5 | 4.2 | 1.6 |
|  | Imagine-VR |  | 4.3 | 1.4 | 4.0 | 1.4 |
|  | Imagine and Embodied-VR |  | 3.9 | 1.3 | 4.4 | 1.5 |
| Liking | Imagine-IV |  | 5.8 | 1.0 | 6.4 | 1.0 |
|  | Imagine-VR |  | 5.5 | 1.0 | 6.1 | 0.6 |
|  | Imagine and Embodied-VR |  | 5.46 | 1.2 | 6.2 | 0.7 |
| Vividness | Imagine-IV |  | 3.8 | 1.7 | 4.5 | 1.5 |
|  | Imagine-VR |  | 3.5 | 1.3 | 4.1 | 1.5 |
|  | Imagine and Embodied-VR |  | 3.3 | 1.5 | 3.9 | 1.2 |

To find the between condition influence on each of the mediators, PROCESS macro ran four logistic regressions of the condition on each of the future self domains at T1 controlling for the future self domains at T0 and VVIQ scores. The conditions had no effect on the mediating future self domains at T1.

**Condition on Intention-Behavior Rate Controlling for Future Self-Domains**

The future self domains at T1 and condition were regressed upon the intention-behavior rate while controlling for baseline future self domains and VVIQ. This analysis shows the same pattern of results as the main analysis with the positive effect from connectedness at T1 (*b* = 0.76, *Z* = 2.5, *p* = .020, *OR* = 2.13) and the negative effect from similarity at T1 (*b=* -0.71, *Z* = -2.32, *p =* .036, *OR =* 0.49) shows runs counter to the hypothesized direction.

**Figure 3.**

*Mediation Results*

Connected T1

-0.31

0.76**

-0.71*

Similarity T1

-0.22

Imagine and Embodied-VR

0.87

Intention-Behavior Rate

-0.43

0.49

-0.15

Vividness T1

-0.18

Liking T1

Total effect from Imagine and Embodied-VR to Intention-Behavior Rate: *B =* 0.87*, p =* .294

Indirect effect via Connected T1*: b* = -0.24, 95% bootstrap CI [-1.79, 0.42]

Indirect effect via Similarity T1*: b* = -0.22, 95% bootstrap CI [-1.49, 0.56]

Indirect effect via Vividness T1*: b* = -0.21, 95% bootstrap CI [-0.59, 0.59]

Indirect effect via Liking T1*: b* = 0.03, 95% bootstrap CI [-0.59, 0.59]

*Note:* The paths from Imagine-IVand Exposure VR to the mediators are unstandardized, while the direct and indirect paths to the Intention-Behavior Rate are expressed in log-odds. This model controls for baseline future self domains and imaginative ability. Asterisks represent significance levels where **^●^** *p <* .10***,*** * *p* < .05, ** *p* < .01, *** *p* < .001.

**Table 2.**

*Future Self Domains Predicting Intention-Behavior Rate by Condition*

|  | Conditional Model | Mediation Model |
| --- | --- | --- |
| *Direct Effect* |  |  |
| Imagine-IV | 1.37 (2.97) | 0.47 (3.37) |
| Imagine-VR | -0.08 (1.58) | -0.61 (0.85) |
| Imagine and Embodied-VR | 0.3 (1.67) | 0.87 (0.83) |
| Connectedness T1 |  | 0.76 (0.30)** |
| Similarity T1 |  | -0.71 (0.31)* |
| Liking T1 |  | -0.15 (0.47) |
| Vividness T1 |  | 0.49 (0.32) |
| *Control Variables* |  |  |
| Connectedness T0 | -0.1 (0.27) | -0.33 (0.23) |
| Similarity T0 | -0.14 (0.33) | 0.14 (0.27) |
| Liking T0 | -0.16 (0.37) | -0.20 (0.29) |
| Vividness T0 | 0.45 (0.27)* | 0.20 (0.29) |
| VVIQ | -0.01 (0.04) | 0.0 (0.03) |

*Note:* All values are odds ratios with standard errors in brackets. Asterisks represent significance values at * *p* < .05, ** *p* < .01, *** *p* < .001.

**Conclusion**

We removed the 15 people who misremembered their behavioral intention at T2. Excluding these participants did not change any results besides future self vividness at baseline being a predictor of the behavior-intention rate.
