# Supplementary material for: Feeling connected but dissimilar to one’s future self reduces the intention-behavior gap
Source: PLoS One. 2024 Jul 23;19(7):e0305815. doi: 10.1371/journal.pone.0305815 (PMC11265703; doi:10.1371/journal.pone.0305815)
Supplement: Attachment — Submitted filename: Response to Reviewers Letter.docx [file pone.0305815.s009.docx]

Response to Reviewers Letter

I made an error in putting 2018 instead of 2019 as the start of the data collection in line 154 of the manuscript. I made the error because we did not put the start of data collection date in earlier versions of this manuscript and my I misremembered the start date of the study. The reviewer correctly pointed out that the date of data collection is before the date of the ethics approval. I thank the reviewer for finding this error and hope you will still accept this paper for submission.

Thank you,

Benjamin Ganschow

Here is the rejection letter:

PONE-D-23-13476
Feeling Connected But Dissimilar to One’s Future Self Reduces the

Intention-Behavior Gap
PLOS ONE

Dear Dr. Ganschow,

Thank you very much for submitting your manuscript to PLOS ONE, and for responding to our recent requests regarding your submission. After careful evaluation, we have decided that your submission does not meet our publication criteria and must be rejected.

Based on our evaluation of your manuscript and the information you provided, we do not feel that your submission meets our ethical requirements for human subjects research submissions. PLOS ONE requires that research meets all applicable standards for the ethics of experimentation and research integrity (<http://journals.plos.org/plosone/s/human-subjects-research>). We reserve the right to reject any submission that does not meet our internal ethical standards, which in some cases are more stringent than local ethical standards.

We recognize that you obtained ethical approval from your local ethics committee after you began the study: your application for ethics approval document is dated 25.10.2019, while participants were recruited in December to February 2018. For human subjects research, PLOS ONE requires that authors obtain ethics committee approval before beginning the study, and we do not accept retrospective ethical approval.

As a result of these concerns we cannot consider the manuscript for publication at PLOS ONE. I am sorry that we cannot be more positive on this occasion, but hope that you understand the reasons for this decision.

Kind regards,

Jayati Bhattacharyya, PhD
Editorial Office
PLOS ONE
